# Reduced Incidence of Cardiac Arrhythmias in Walkers and Runners

**DOI:** 10.1371/journal.pone.0065302

**Published:** 2013-06-07

**Authors:** Paul T. Williams, Barry A. Franklin

**Affiliations:** 1 Life Sciences Division, Lawrence Berkeley National Laboratory, Berkeley, California, United States of America; 2 Preventive Cardiology and Cardiac Rehabilitation, William Beaumont Hospital, Beaumont Health Center, Royal Oak, Michigan, United States of America; University of Bath, United Kingdom

## Abstract

**Purpose:**

Walking is purported to reduce the risk of atrial fibrillation by 48%, whereas jogging is purported to increase its risk by 53%, suggesting a strong anti-arrhythmic benefit of walking over running. The purpose of these analyses is to compare incident self-reported physician-diagnosed cardiac arrhythmia to baseline energy expenditure (metabolic equivalent hours per day, METhr/d) from walking, running and other exercise.

**Methods:**

Proportional hazards analysis of 14,734 walkers and 32,073 runners.

**Results:**

There were 1,060 incident cardiac arrhythmias (412 walkers, 648 runners) during 6.2 years of follow-up. The risk for incident cardiac arrhythmias declined 4.4% per baseline METhr/d walked by the walkers, or running in the runners (P = 0.0001). Specifically, the risk declined 14.2% (hazard ratio: 0.858) for 1.8 to 3.6 METhr/d, 26.5% for 3.6 to 5.4 METhr/d, and 31.7% for ≥5.4 METhr/d, relative to <1.8 METhr/d. The risk reduction per METhr/d was significantly greater for walking than running (P<0.01), but only because walkers were at 34% greater risk than runners who fell below contemporary physical activity guideline recommendations; otherwise the walkers and runners had similar risks for cardiac arrhythmias. Cardiac arrhythmias were unrelated to walking and running intensity, and unrelated to marathon participation and performance.

**Conclusions:**

The risk for cardiac arrhythmias was similar in walkers and runners who expended comparable METhr/d during structured exercise. We found no significant risk increase for self-reported cardiac arrhythmias associated with running distance, exercise intensity, or marathon participation. Rhythm abnormalities were based on self-report, precluding definitive categorization of the nature of the rhythm disturbance. However, even if the runners’ arrhythmias include sinus bradycardia due to running itself, there was no increase in arrhythmias with greater running distance.

## Introduction

Atrial fibrillation (AF)/flutter is the most common cardiac arrhythmia in endurance-trained athletes [1], and AF increases the risk for stroke, bleeding from anticoagulants, and reduced exercise tolerance [2]–[4]. Obesity, treated hypertension, coronary and valvular heart disease, left ventricular enlargement, and heart failure are known risk factors for AF [2], [5].

There is concern that habitual exercise could increase the frequency of AF as well as other rhythm disturbances such as premature atrial (PAC) and ventricular (PVC) contractions. There is no greater difference in the relative advantage of walking over running than their purported different effects on the risk of AF. The Physicians Health Study reported a 53% greater risk in men who jogged five to seven days per week vis-à-vis non-joggers [6]. In contrast, the Cardiovascular Health Study reported a 48% lower risk in men and women who walked ≥60 blocks per week at >3 mph as compared to those who walked ≤4 blocks per week at <2 mph [7]. It is hypothesized that the high-intensity training of long-distance runners and other endurance athletes results in left ventricular hypertrophy, which increases the risk of AF [8]–[14]. Theoretically at least, atrial and ventricular arrhythmias in marathoners and participants in extreme endurance events could arise due to transient acute volume overload of the atria and right ventricle, and reductions in right ventricular ejection fraction, causing patchy myocardial fibrosis in the atria, interventricular septum, and right ventricle [15], [16]. Walking is purported to reduce risk factors for AF in the absence of left ventricular hypertrophy [7]. Age and sex may affect the relationship between exercise and AF. Atrial fibrillation increased with increasingly vigorous exercise in men <50 years but not older men in the Physicians Health Study [6], whereas all subjects in the Cardiovascular Health Study were ≥65 years [7]. The majority of veteran endurance athletes who develop AF are male, consistent with sex differences in atrial and ventricular remodeling and other AF risk factors [17].

This investigation examines the relationship of walking and running to self-reported physician-diagnosed heart rhythm disturbances (cardiac arrhythmias) in a very large sample of runners and walkers (n = 46,807), of whom 59% are women, 41% are men, 60% are <50 years, and 40% are ≥50 years of age. While lacking the specificity of clinically-diagnosed AF, the analyses are well-powered to detect a 53% risk increase from jogging, a 48% risk decrease from walking, and the significance of their difference, even assuming a 50% dilution of AF events due to other heart rhythm disturbances.

## Methods

### Ethics Statement

The study protocol was reviewed and approved by the Human Subjects Committee at Lawrence Berkeley National Laboratory for the protection of human subjects, and all subjects provided a signed statement of informed consent.

These prospective analyses were derived from the 2006 partial re-survey of the National Runners’ Health Study II and the National Walkers’ Health Study [18]–[22]. The re-survey was conducted to provide a base population of approximately 50,000 runners and walkers for consideration in a future clinical trial, rather than an attempt to obtain a high response rate from a more manageable sample size. The participants completed a two-page baseline and four-page follow-up questionnaire on running and walking history, body weight and waist circumference, diet (vegetarianism and the current weekly intakes of alcohol, red meat, and fruit), cigarette use, and history of chronic diseases. Cardiac arrhythmias were based on the self-reported physician-diagnosed “heart rhythm disturbance”, and the year of diagnosis relative to the baseline survey date.

Walking and running distances were reported in miles walked or run per week. Non-running energy expenditures in the runners, and non-walking energy expenditures in the walkers, were calculated using previously published metabolic equivalent (MET) tables [23], where one MET is the energy expended sitting at rest (3.5 ml O_2_•kg^−1^•min^−1^) [23]. Physical activities that expended 3 to 6 METs were classified as moderate-intensity exercise, >6 METs as vigorous, and <3 METs as light [24]. Distance-based calculations of metabolic equivalent hours per day (METhr/d) walked converted reported distance into duration (i.e., distance/mph) and then calculated the product of the average hours walked per day and the MET value for the reported pace [23]. Running MET values were calculated as 1.02 MET•hours per km [19]. Distance run has been shown to correlate strongly (r = 0.89) on repeated questionnaires [25]. Distance run and walked have been shown to provide a superior metric for estimating METhr/d run and walked than their traditional time-based estimates [18], [19], [26]. Strength exercise included lifting weights, circuit training, resistance or strength training, crunches, abdominal exercise, push-ups, pull-ups, sit-ups, leg lifts, upper body exercise, and varied calisthenics.

Statistical analyses were performed using JMP (SAS institute, Cary NC, version 5.1) and Stata (StataCorp LP, College Station TX, version 11). Cox proportional hazard analyses were used to estimate the hazard rate per METhr/d of running, walking, and other vigorous, moderate, and light intensity exercise. Covariates included adjustments for age (age and age^2^), sex, education, intakes of meat, fruit, and alcohol, and current smoking status. Linear contrasts were used to compare regression slopes for running, walking, and other exercise.

## Results

Of the 49,005 potentially eligible subjects, we excluded 1076 subjects for pre-existing coronary heart disease, and 1,122 subjects for prior arrhythmias. There were 520 incident events among the remaining 19,044 men and 540 incident events among the remaining 27,763 women during the average 6.2-year follow-up. Their characteristics are presented in Table 1. As expected, walkers and runners who reported an incident event were older than those who did not. They also tended to walk less if they were walkers, and run less if runners. Body mass index (BMI) was slightly greater for those reporting events. There were 35.4% of the walkers and 52.4% of the runners who reported walking or running ≥10 years, 19.2% and 37.2% who reported ≥15 years, respectively, and 9.9% and 20.8% who reported ≥20 years, respectively.

**Table 1:** Characteristics of walkers and runners by self-reported physician-diagnosed cardiac arrhythmia.

	Walkers		Runners	
	Events	Non-events	Events	Non-events
N	412	14,322	648	31,425
Male (%)	26.0	18.6	63.7	50.5
Age (years)	59.5±12.6	54.0±12.1	50.8±12.4	44.3±11.2
Education (years)	15.6±2.6	15.5±2.6	17.0±2.2	16.6±2.4
Smokers (%)	2.9	3.6	0.8	1.5
Meat (serving/d)	0.42±0.35	0.39±0.35	0.36±0.35	0.36±0.37
Fruit (pieces/d)	1.7±1.2	1.7±1.2	1.6±1.1	1.5±1.1
Alcohol (g/d)	6.1±10.5	5.8±10.2	9.5±12.4	7.8±11.3
Exercise energy expenditure (METhr/d)				
Running			4.7±3.1	5.0±3.1
Walking	1.9±1.6	2.2±1.6		
Other vigorous	1.4±2.7	1.5±3.1	2.1±3.5	1.9±3.3
Other moderate	0.4±1.3	0.4±1.3	0.8±1.6	0.8±1.7
Other light	0.1±0.5	0.0±0.4	0.0±0.6	0.0±0.3
Strengthening	0.2±0.9	0.2±0.8	0.5±1.0	0.5±1.3
Non-strengthening	1.7±3.0	1.7±3.2	2.5±3.9	2.2±3.5
Pace (m/s)	1.6±0.3	1.6±0.3	3.0±0.5	3.1±0.5
Walking frequency (walks/d)	0.8±0.4	0.8±0.5		
Longest walk (km)	5.7±3.2	6.5±4.0		
10-km performance (m/s)			3.5±0.6	3.6±0.6
Marathons (# in 5 years)			1.8±4.3	1.7±4.0
Years run or walked	9.1±8.5	8.9±8.3	14.4±10.2	11.9±8.6

Percent or mean ±SD. METhr/d: Metabolic equivalent hours per day.

**Figure 1:**
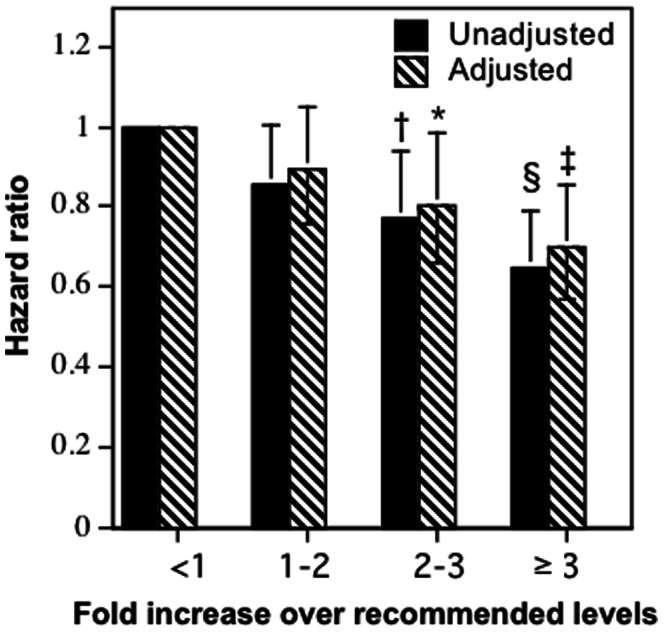
Reduction in the risks for incident cardiac arrhythmias per METhr/d for walking and running energy expenditure combined relative to the least active walkers and runners. Adjustment for BMI and hypertension treatment had little effect on these risk reductions. Energy expenditure (X-axis) is categorized in terms of the upper limit of the minimum recommended physical activity levels (750 MET**⋅**min/wk = 1.8 METhr/d [10], [18]), e.g., 1 to 2-fold higher activity covers from 1.8 to 3.6 METhr/d, 2- to 3-fold covers from 1.6 to 3.4 METhr/d etc. Error bars represent 95% confidence intervals. Significant levels relative to <1.8 METhr/d coded: * P<0.05; † P<0.01, ‡ P<0.001, and ‡ P<0.0001. The superscript § means that the risk for runners who ran ≥3-fold was significantly less than those who ran <1-fold the recommended level.

The risk for arrhythmias declined 4.8% per METhr/d run or walked (95% CI: 7.2% to 2.3%, P = 0.0001). Figure 1 (runners and walkers combined) suggests a relatively linear decline in relative risks (hazard ratios) by reported total METhr/d walked by walkers and run by runners. Specifically, relative to those who ran or walked <1.8 METhr/d (750 MET•min/wk, the upper limit of the minimum exercise level recommended for health), the risk for incident cardiac arrhythmias was reduced 14.2% by exceeding the recommendations by 1- to 2-fold (P = 0.08), 26.5% by exceeding the recommendations by 2- to 3-fold (P = 0.02), and 31.7% by exceeding the recommendations by ≥3-fold (P = 7.3×10^−5^). Adjustment for BMI and hypertension treatment had little effect on these risk reductions.

### Walking

Table 2 presents the proportional hazard analyses for all subjects and subjects under 50 years old. Energy expended during walking was associated with a reduced risk of cardiac arrhythmias (11.8% reduction per METhr/d, P = 0.0001) particularly in younger men and women (21.0% reduction, P = 0.0004), but also significantly in those ≥50 years old (9.6% reduction, P = 0.007 not displayed). The risk reduction was only slightly diminished by adjusting for baseline BMI and hypertension treatment for the total sample (9.8% reduction per METhr/d after adjustment, P = 0.002), and for the younger (19.1% reduction, P = 0.002) and older subsets (7.6% reduction, P = 0.04 not displayed). Sex did not appear to affect the reduction in risk per METhr/d walked (male vs. female: 15.1% vs. 10.3% reduction per METhr/d, P = 0.58 for difference).

**Table 2:** Hazard ratios (95% confidence intervals) of incident self-reported physician- diagnosed cardiac arrhythmias vs. exercise energy expenditure during 6.2-year follow-up.

	No adjustment for BMI and hypertension treatment		Adjusted for BMI and hypertension treatment	
	All ages	<50 years	All ages	<50 years
Sample size (N)	46,807	28,026	46,250	27,777
Incident cases	1060	408	1052	407
Education (years)	1.038†	1.064†	1.039†	1.066†
	(1.013, 1.063)	(1.020, 1.110)	(1.015, 1.065)	(1.022, 1.112)
Smoking (0,1)	0.865	1.175	0.863	1.155
	(0.513, 1.353)	(0.582, 2.098)	(0.512, 1.350)	(0.572, 2.063)
Red Meat (servings/day)	1.032	1.224	1.000	1.186
	(0.871, 1.224)	(0.937, 1.572)	(0.841, 1.188)	(0.905, 1.528)
Fruit (pieces/day)	1.005	1.081	1.007	1.083
	(0.953, 1.057)	(0.985, 1.181)	(0.956, 1.060)	(0.987, 1.183)
Alcohol (g/day)	1.003	0.995	1.002	0.995
	(0.998, 1.008)	(0.983, 1.005)	(0.997, 1.007)	(0.984, 1.005)
Runners (0,1)	0.732†	0.506‡	0.828	0.569†
	(0.579, 0.925)	(0.341, 0.758)	(0.648, 1.058)	(0.376, 0.867)
Energy expenditure (METhr/d)				
Running	0.965†	0.969	0.972*	0.975
	(0.940, 0.992)	(0.932, 1.006)	(0.946, 0.999)	(0.937, 1.012)
Walking	0.882§	0.790‡	0.902†	0.809†
	(0.825, 0.942)	(0.683, 0.904)	(0.843, 0.964)	(0.699, 0.927)
Other vigorous	1.020*	1.020	1.021*	1.021
	(1.002, 1.037)	(0.991, 1.046)	(1.003, 1.038)	(0.992, 1.047)
Other moderate	0.995	1.002	0.967	1.003
	(0.955, 1.031)	(0.936, 1.058)	(0.957, 1.033)	(0.936, 1.059)
Other light	1.074	1.176	1.076	1.178
	(0.955, 1.151)	(0.972, 1.312)	(0.958, 1.152)	(0.974, 1.314)
BMI			1.031‡	1.030*
			(1.014, 1.049)	(1.002, 1.057)
Hypertension			1.110	0.978
			(0.915, 1.347)	(0.529, 1.649)
Comparison between coefficients				
Running vs. walking	P = 0.01	P = 0.006	P = 0.04	P = 0.01
Running vs. other vigorous exercise	P = 0.001	P = 0.03	P = 0.003	P = 0.05
Walking vs. other moderate exercise	P = 0.002	P = 0.002	P = 0.01	P = 0.007

Significance levels coded: *P≤0.05;

† P≤0.01;

‡ P≤0.001;

§ P≤0.0001.

**Figure 2:**
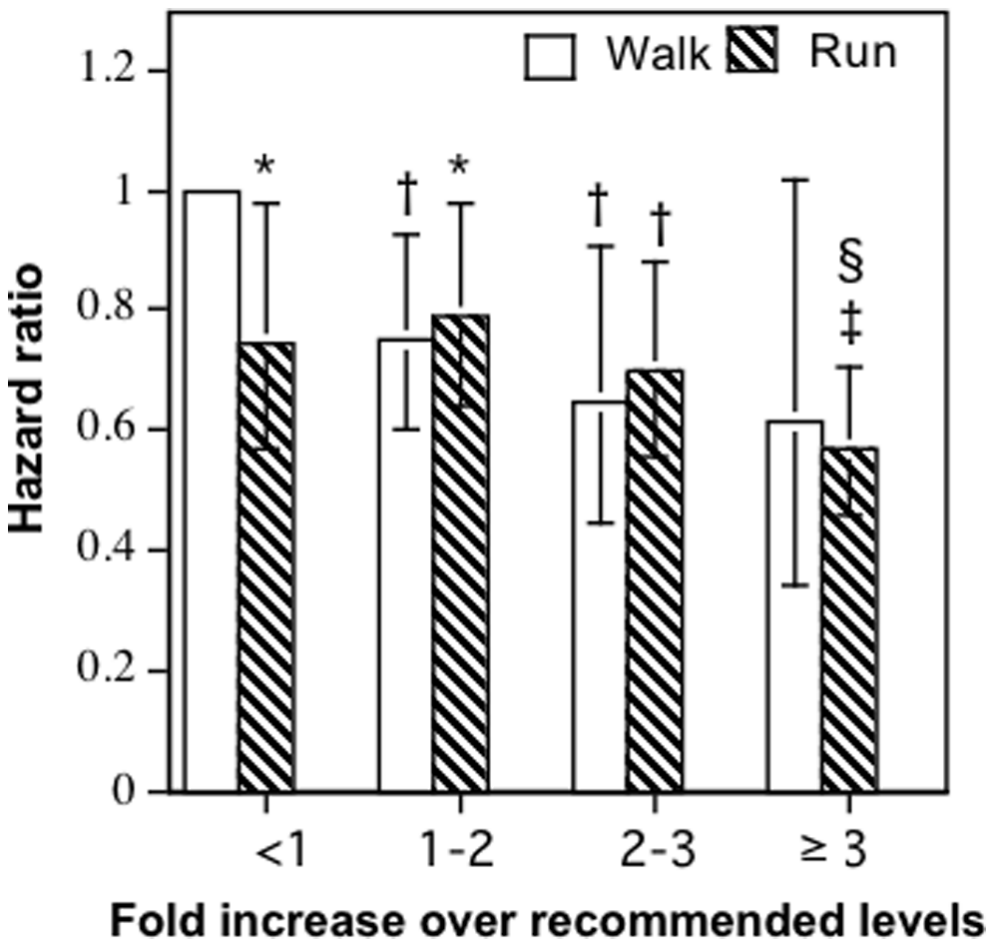
Reduction in the risks for incident cardiac arrhythmias per METhr/d energy expended by walking and running separately relative to the least active walkers. Energy expenditure (X-axis) is categorized in terms of the upper limit of the minimum recommended physical activity levels (750 MET**⋅**min/wk = 1.8 METhr/d [10], [18]), e.g., 1 to 2-fold higher activity covers from 1.8 to 3.6 METhr/d, etc. Error bars represent 95% confidence intervals. Significant levels relative to the least active walkers coded: * P<0.05; † P<0.01, and ‡ P<0.0001. The superscript § means that the risk for runners who ran ≥3-fold was significantly less than those who ran <1-fold the recommended level (P<0.05).

Figure 2 shows that relative to walkers who walked <1.8 METhr/d, those who exceeded the guideline levels by 1- to 2-fold reduced their risk by 25.3% (P = 0.007), 2- to 3-fold by 35.5%, and ≥3-fold by 38.5% (P = 0.06). Other analyses (not displayed) showed that when adjusted for METhr/d walked, there were some indications that the risk for cardiac arrhythmias decreased with the distance of the longest walk (all ages: P = 0.04; <50 years old: P = 0.10), and frequency of walks per week that were longer than 10 minutes duration (all ages: P = 0.33; <50 years old: P = 0.03), but not usual walking intensity (all ages: P = 0.21; <50 years old: P = 0.69).

### Running

Table 2 shows that the energy expended during running was associated with a reduced risk for incident cardiac arrhythmias (3.5% per METhr/d, P = 0.01) with little difference in the effect in those <50 years (3.1%, P = 0.10) and ≥50 years old (3.3%, P = 0.09 not displayed). The risk reduction was only slightly diminished by adjusting for baseline BMI and hypertension treatment for the total sample (2.8% per METhr/d, P = 0.04). Women demonstrated a significantly greater risk reduction than men (men vs. women: 1.4% vs. 7.2% reduction per METhr/d, P = 0.05 for difference, not displayed), which was diminished slightly when adjusting for BMI and hypertension treatment (men vs. women: 1.3% vs. 6.5% reduction per METhr/d, P = 0.07). Figure 2 shows that relative to runners who ran <1.8 METhr/d, those that exceeded the guideline levels by 1- to 2-fold increased their risk by 6.3% (P = 0.64), 2- to 3-fold reduced their risk by 6.6% (P = 0.63), and ≥3-fold by 25.7% (P = 0.04). Other analyses (not displayed) showed that when adjusted for METhr/d run, the runners’ risk of cardiac arrhythmia was unrelated to running intensity (all ages: P = 0.77; <50 years old: P = 0.77), the number of marathons completed during the previous five years (all ages: P = 0.62; <50 years old: P = 0.74), the best marathon performance times (all ages: P = 0.48; <50 years old: P = 0.66), and 10-km performance times (all ages: P = 0.21; <50 years old: P = 0.25). Most importantly, we found no effect of years run on the risk for cardiac arrhythmias, either as a simple additive effect with METhr/d run (P = 0.69), or as an interaction with METhr/d run (P = 0.79).

The reduction in risk per METhr/d was significantly less for running in runners than walking in walkers (Table 2); however, this was due entirely to walkers who exercised below contemporary physical activity guideline levels having a 34% greater risk of cardiac arrhythmias than runners who exercised below these recommendations (Figure 2). The walker-runner difference in cardiac arrhythmia risk was significant below the guideline levels (<1/8 METhr/d, walkers 34% higher risk than runners, P = 0.03), but not for 1- to 2-fold (walkers 6% lower risk, P = 0.62), 2- to 3-fold (walkers 7.5% lower risk, P = 0.68), and ≥3-fold the guideline levels (walkers 8% greater risk, P = 0.78).

### Other Exercise

Table 2 shows that other vigorous exercise was associated with a slight increase in the risk of cardiac arrhythmias, which remained significant when adjusted for BMI and hypertension treatment. The effects on the risk of cardiac arrhythmias differed significantly between running and other vigorous exercise, and between walking and other moderate exercise. The increased risk associated with other exercise appeared to be due to non-strengthening exercises (P = 0.05), rather than strengthening exercises (P = 0.68, analyses not displayed).

## Discussion

Our analyses show that the risk of incident arrhythmias decreased significantly per METhr/d walked, and that the risk reduction was most pronounced in those <50 years. Walkers that included longer walks as part of their exercise regimen, and younger men and women who walked more frequently, were less likely to report arrhythmias. For runners, the reduction in risk per METhr/d run was also significant, and significantly less than that observed for walking, but only because inadequately exercising walkers (i.e., below guideline levels) were at 34% greater risk for arrhythmias than inadequately active runners. We found no evidence that the incidence of self-reported, physician diagnosed cardiac arrhythmias increased in association with greater marathon participation or performance, greater cardiorespiratory fitness as measured by 10 km performance times, or usual walking or running intensity. The majority of the runners had run over 10 years, and over 20% had run over 20 years, so there was ample opportunity for long-term physiological changes to have occurred.

Our results are consistent with the dose-response relationships between greater physical activity and reduced risk for AF in the Cardiovascular Health Study [7]. These investigators reported a graded reduction in the risks for AF in 5446 adults ≥65 years of age, i.e., 25%, 22%, and 36% reductions for the 3^rd^, 4^th^, and 5^th^ highest quintiles of physical activity relative to the least active quintile [7]. Both walking pace and distance exhibited a graded reduction in AF risk. In contrast to our findings, the risk of AF was not decreased by high intensity exercise, and a U-shaped relationship between exercise intensity and AF was noted.

Our results technically support the hypothesis that the risk reduction may be less for running than walking, but this was entirely due to the 34% excess risk in inadequately active walkers. At 1- to 2-fold, 2- to 3-fold, and ≥3-fold increases over recommended activity levels the risks for cardiac arrhythmias were comparable for walking and running. Our analyses did not show that running longer distances, or at greater intensities, increases the prevalence of self-reported cardiac arrhythmias. Our results, therefore, are in contrast to the Physicians’ Health Study conclusion that men who are vigorously active, particularly those who jogged, were at greater risk for developing AF [6]. They reported that the association with vigorous exercise or jogging was primarily among those who reported 5 to 7 days/week of structured physical activity. Specifically, men who jogged 1 to 2, 3 to 4, and 5 to 7 days per week were at 3%, 30% and 53% greater risk for AF compared to non-joggers. Incident AF was not significantly related to the frequency of vigorous exercise when joggers were excluded from their analyses, and there was no significant relationship to regular cycling, swimming or racquet sports. Admittedly, the discrepancy could be due to the Physicians’ Health Study’s greater specificity and more rigorous diagnoses of AF. However, we would not expect the lack of specificity and reporting biases of the current study to differ between walkers (where we show consistency with Cardiovascular Health Study results) and runners (inconsistent with the Physicians’ Health Study results).

The evidence relating AF to vigorous exercise is stronger for case control comparisons than prospective studies. Case-control studies show greater odds for AF in orienteers [15], endurance sport participants [8], [14], [27] marathon runners [28], and cyclists [10], [29] than non-athletic controls. Prospectively, lone AF was reported to be 4-fold greater in 252 marathon runners (0.43/100) as compared with 305 sedentary men (0.11/100) in a follow-up cohort study [28]. The risk for lone AF has also been associated with moderate and vigorously intense occupational physical activity [27]. Several studies have reported no significant relationship between lone AF and physical activity [30], [31].

Our results suggested a greater risk reduction per METhr/d walked for men and women <50 years old than among older exercisers. Importantly, the decision to subdivide our population at age 50 was made a priori in order to correspond to the Physicians’ Health Study report [6]. That study found a significant interaction between age (≥50 vs. <50 years old) and being vigorously active 5 to 7 days a week (P = 0.02), i.e., 20%, 5% and 74% greater risk of AF for exercising vigorously 1 to 2, 3 to 4, and 5 to 7 days per week, respectively, relative to zero days in those under 50. The investigators suggested that because parasympathetic activity is reduced with age, parasympathetic enhancement from vigorous exercise would likely diminish in older men. Because older individuals were more likely to have underlying structural heart disease, the proportion having AF with no known etiology (i.e., lone) would also likely diminish. These factors may contribute to the greater risk reduction we observed in younger vis-à-vis older walkers.

Our analyses also revealed significantly greater anti-arrhythmic risk reductions for running vs. other vigorous exercise, and for walking vs. other moderate exercise. Whereas energy expenditure for walking and running were calculated from reported distances walked or run per week, other exercise was calculated from the reported exercise duration (time). We previously reported that time-based estimates of running and walking exhibit much weaker relationships to BMI and regional adiposity cross-sectionally than their distance-based calculations [18], [19], [26]. Presumably, the greater inaccuracy and biases associated with time-based estimation would also apply to other exercise, for which there is no distance-based alternative. Therefore, the differences we observed may relate in large part to the methods of estimation, rather than physiological effects of non-running vigorous exercise and non-walking moderate exercise. For running in particular, Aizer et al. suggested that the pro-AF effects should be greater for jogging than for other vigorous exercise because running produces greater enhancement of the parasympathetic nervous system than other exercise types, contrary to what we observed [6].

### Limitations

The primary limitations of these analyses are the lack of medical record verification and specificity for AF, and the study design. Our documented rhythm abnormalities were based on self report so it is impossible to determine the nature of the rhythm disturbance. AF/flutter is the most common cardiac arrhythmia in endurance-trained athletes [1], and should represent the majority of the events reported. Nevertheless, benign sinus bradycardia or prolonged PR interval, QRS, and QT duration characteristic of endurance-training could be misinterpreted by the participant as a heart rhythm disturbance, and contribute to the runners’ smaller risk reduction. However, the lack of an increase in cardiac arrhythmias in higher mileage runners and the lack of a significant difference between higher mileage walkers and runners would be therefore conservative, in that they would overestimate clinically important arrhythmias in the runners, thereby increasing the incidence of clinically important arrhythmias in the highest mileage group and the difference between walkers and runners. In addition, the analyses are likely to include mostly self-reported symptomatic arrhythmic events (e.g., palpitations or hemodynamic symptoms) that are like to have been preceded by asymptomatic episodes of supraventricular arrhythmias, and periods of asymptomatic atrial flutter and fibrillation [32]. The study was not designed as a prospective follow-up study, but rather samples were re-surveyed to provide approximately 50,000 runners and walkers for the current analysis. Nevertheless, we would anticipate that the biological process relating exercise to cardiac arrhythmias in this sample should be the same as in the general population. The re-contact of runners and walkers for the follow-up survey followed identical protocols, and therefore selection bias is unlikely to account for the walker-runner differences. The National Runners’ and Walkers’ Health Studies are unique in being the only large cohorts specifically selected to study the health benefits of moderate intensity (walking) and vigorous intensity exercise (running). We do not believe that the declining incidence of cardiac arrhythmias with greater METhr/d walked or run was due to fewer opportunities for diagnosis in the more athletic men. The Health Professional Study reported that their more vigorously active participants had more routine medical check-ups than less active men [33] and there was no difference in routine medical check-up by activity level in the Nurses Health Study [34].

In conclusion, these data suggest that walking and running affect the incidence of cardiac arrhythmias similarly, except for substantially higher risk in walkers who fail to meet contemporary physical activity guideline levels. Reduced AF may represent one of the mechanisms by which moderate physical activity, and walking in particular, reduces cardiovascular disease risk [35] and stroke [36]. Hypertension, high cholesterol, and diabetes are also reduced in association with walking distance [37]. Higher doses of running have been shown prospectively to reduce the risks of coronary heart disease through at least 9 km/d [38]. Moreover, Figure 1 clearly demonstrates the added anti-arrhythmic benefits from substantially exceeding the minimum guideline activity levels currently recommended for health [23], [39]. However, these data do not negate the importance of careful monitoring of runners with symptoms or at high risk for arrhythmias [40].
